# Progression of urothelial carcinoma in situ of the urinary bladder: a switch from luminal to basal phenotype and related therapeutic implications

**DOI:** 10.1007/s00428-018-2354-9

**Published:** 2018-04-13

**Authors:** Isabella Barth, Ursula Schneider, Tobias Grimm, Alexander Karl, David Horst, Nadine T. Gaisa, Ruth Knüchel, Stefan Garczyk

**Affiliations:** 1 Institute of Pathology, University Hospital RWTH Aachen, Pauwelsstrasse 30, 52074 Aachen, Germany; 2 Department of Urology, LMU Munich University, Munich, Marchioninistraße 15, 81377 Munich, Germany; 3 Institute of Pathology, LMU Munich University, Munich, Thalkirchner Str. 36, 80337 Munich, Germany

**Keywords:** Bladder Cancer, Carcinoma in situ (CIS), Molecular subtypes, Her2/neu, Estrogen receptor Beta (ERβ), Targeted therapy

## Abstract

The stratification of bladder cancer into luminal and basal tumors has recently been introduced as a novel prognostic system in patient cohorts of muscle-invasive bladder cancer or high-grade papillary carcinomas. Using a representative immunohistochemistry panel, we analyzed luminal and basal marker expression in a large case series (*n* = 156) of urothelial carcinoma in situ (CIS), a precancerous lesion that frequently progresses to muscle-invasive disease. The majority of CIS cases was characterized by a positivity for luminal markers (aberrant cytokeratin (CK) 20 85% (132/156), GATA3 median Remmele score (score of staining intensity (0–3) multiplied with percentage of positive cells (0–4)): 12, estrogen receptor (ER) β Remmele score > 2: 88% (138/156), human epidermal growth factor receptor 2 (Her2) Dako score 3+ 32% (50/156), Her2 Dako score 2+ 33% (51/156)), and marginal expression of basal markers (CK5/6+ 2% (3/156), CK14+ 1% (2/156)). To further investigate phenotypic stability during disease progression, we compared 48 pairs of CIS and invasive tumors from the same biopsy. A highly significant loss of luminal marker expression (*p* < 0.001) was observed in the course of progression whereas an increase of basal marker expression (*p* < 0.01) was noted in the invasive compartment. Importantly, 91% of CIS cases demonstrated a positivity for at least one of the two predictive markers Her2 and ERβ, indicating that the analysis of Her2 and ERβ may help to identify CIS-patient subgroups prone to more efficient targeted treatment strategies. Larger prospective and biomarker-embedded clinical trials are needed to confirm and validate our preliminary findings.

**Electronic supplementary material:**

The online version of this article (10.1007/s00428-018-2354-9) contains supplementary material, which is available to authorized users.

## Introduction

Cancer arising from the urothelium of the bladder is estimated to be the fourth most common malignancy in American men, with an estimated 80,000 new cases in 2017 in the USA [1]. Most bladder carcinomas present as non-muscle-invasive, low-grade papillary carcinomas and are associated with an excellent prognosis. These tumors recur frequently but rarely progress to muscle-invasive disease. In contrast, muscle-invasive bladder cancer (MIBC) is associated with an unfavorable prognosis (5-year survival < 50%) due to a high risk of local and systemic disease progression [2]. Most MIBCs arise from carcinoma in situ (CIS), a flat, high-grade, superficial urothelial lesion that is characterized by *TP53* mutations and accounts for approximately 10% of all diagnosed bladder tumors [3]. CIS is considered to be an obligate precancerous lesion, while concomitant CIS is associated with a markedly worse prognosis in patients with bladder cancer, a fact that is mirrored in multiple clinical risk scores (Spanish Urological Club for Oncological Treatment (CUETO), European Organization for Research and Treatment of Cancer (EORTC)) [4].

Therapeutic options for urothelial CIS are limited and based on a moderate to low level of evidence. To inhibit disease progression and recurrence, current guidelines recommend transurethral resection, followed by intravesical instillation of Bacillus Calmette-Guérin (BCG) as first-line therapy [5, 6]. BCG instillation, however, has numerous side effects, and a high rate of tumors persist or recur irrespective of treatment [7]. In such cases, a radical cystectomy is usually performed, a procedure with high surgical morbidity [8]. Therefore, the need for new and efficient local therapies as well as reliable prognostic and predictive markers are of upmost clinical importance.

A recent stratification of bladder cancer into two main molecular subtypes with prognostic significance—“luminal” and “basal”—has furthered our understanding of urothelial carcinoma biology [9–12]. Luminal tumors are characterized by the same markers as the differentiated layer of the urothelium, while basal tumors are enriched with squamous markers like CK5 and CK14, typical for undifferentiated basal cells [13]. In MIBC, luminal tumors have a favorable prognosis, while basal carcinomas are associated with advanced cancer stages at diagnosis, with metastatic disease and shorter overall survival [9–12]. Intriguingly, molecular stratification of a large, heterogeneous cohort of early high-grade bladder cancer revealed an inverse correlation between prognosis and molecular subtype, defining the luminal subgroup as the more aggressive one in non-muscle-invasive bladder cancer (NMIBC) [14]. However, only three cases of CIS were included in this study and it remains unclear whether this classification is also applicable in CIS.

Aiming to investigate the role of the recently discovered molecular subtypes of bladder cancer in urothelial CIS, we analyzed luminal and basal marker expression using an established surrogate immunohistochemistry (IHC) panel comprising the luminal markers CK20, GATA3, human epidermal growth factor receptor type 2 (Her2), and estrogen receptor (ER) β as well as the basal markers CK5/6 and CK14 in 156 CIS tissue samples from 132 patients. Furthermore, we compared protein expression of these markers in CIS and corresponding invasive tumor parts of the same patient, in an attempt to better understand the stability of a potential subgroup affiliation in the process of stromal invasion.

Moreover, we included two predictive markers and potential treatment targets in our IHC panel, ERβ and Her2, aiming to evaluate new therapeutic options for urothelial CIS. The significance of ERβ signaling in urothelial cancer proliferation and the feasibility of ER targeting therapies have been recognized in numerous studies [15–17]. Anti-Her2 therapies are established treatment options in cases of *Her2*-amplified breast and gastric cancer and have been investigated and proposed as a therapeutic strategy for MIBC [18–20]. To clarify the molecular background of Her2 expression in urothelial CIS, we performed fluorescence in situ hybridization (FISH) in a large fraction of our CIS cohort. We aimed to examine whether protein expression of Her2 and ERβ may yield conclusive findings on potential therapeutic targets for urothelial CIS.

## Materials and methods

### Patients

One hundred fifty-six samples from 132 patients with urothelial CIS who were treated at the RWTH Aachen University and the LMU Munich University Hospitals between 2004 and 2017 were retrospectively included in this study. Clinicopathological data were obtained and an experienced pathologist (RK) reviewed the histological specimens to confirm the diagnosis. Since it is postulated that flat and papillary lesions develop along different pathways [21], patients with previous or concomitant papillary lesions were excluded from this study. Also, patients with a previous diagnosis of invasion into the lamina propria (≥pT1) were not included. This study was conducted at the University Hospital RWTH Aachen in accordance with the requirements of the institutional review board of the RWTH Aachen University (EK 173/06, EK 291/16), the current version of the Declaration of Helsinki, and the good clinical practice guidelines.

For 48 patients, marker expression in cases with CIS and associated concomitant invasive tumor in the same specimen was analyzed. Thorough selection was performed in order to assure that the examined CIS was the precursor of the matched invasive tumor, limiting the chance of a secondary tumor cell spread from an invasive carcinoma within the adjacent urothelium. Besides the exclusion of high-grade papillary lesions, only specimens with the same localizations were compared, and only patients with unifocal invasion were included.

### Immunohistochemistry

IHC was performed on frozen and formalin-fixed, paraffin-embedded (FFPE) tissue. In 10 cases, the specimens had been stored as frozen samples, which were stained on separate slides. Here, the pre-treatment protocol was 10 min fixation in 4 °C acetone, and for ERβ staining, 30 min fixation in 0.5% formalin, followed by immersion in 0.1% triton/paraformaldehyde.

From 145 FFPE cases of CIS and 48 FFPE cases of concomitant invasive tumor, tissue microarrays (TMAs) were constructed with two punches per case where available [22, 23]. In 84 cases, concomitant normal urothelium from the same time of biopsy was available. Punches of positive and negative controls were placed on the TMAs to reduce experimental variability between staining runs. For pre-treatment, TMA sections (2 μm) were incubated in antigen retrieval solution (PT Link, Dako) at 95 °C for deparaffinization, rehydration, and epitope retrieval. Both slides with cryogenic and slides with FFPE material were then treated with EnVision™ Flex Solution (Dako) for 5 min to block endogenous peroxidase activity. Immunostaining was performed with antibodies specific for CK20, GATA3, ERβ, Her2, CK5/6, CK14, and p53 (Online Resource 1). Subsequently, tissue sections were incubated with a secondary reagent for 15 min, followed by treatment with a horseradish peroxidase-conjugated polymer (Dako) for 20 min. The peroxidase reaction was visualized with DAB+ Substrate Chromogen System (Dako). The sections were then counterstained with Mayer’s hematoxylin.

The percentage of cells positive for markers with a cytoplasmic protein reactivity (CK20, CK5/6, CK14) and for p53 was evaluated. p53 accumulation due to mutation was diagnosed if over 20% of cells showed intense nuclear staining [24]. Only nuclear positivity for ERβ and GATA3 was considered relevant and was assessed with an adapted semi-quantitative immunoreactive score, as described by Remmele and Stegner, multiplying a score for nuclear staining intensity of positive cells (0 = negative, 1 = weak, 2 = moderate, 3 = strong) with the percentage of stained cells (0 = 0, 1 < 10%, 2 = 10–50%, 3 = 51–80%, 4 > 80%). A commonly used threshold considers a cancer “positive” when the Remmele Score is 3–12 [25]. Her2 protein expression was graded according to the DAKO score, an established diagnostic tool in breast cancer, combining staining intensity and percentage of stained cells in a semi-quantitative score from 0 to 1 (negative), 2 (moderate), to 3 (positive, overexpressed) [26]. All stained specimens were manually scored by an experienced pathologist (RK), who was blinded to patient identity.

### Fluorescence in situ hybridization

FISH to identify *Her2* gene amplification was performed according to current ASCO recommendations using the Zyto*Light* SPEC ERBB2/CEN 17 Dual Color Probe kit (Zytovision) on 126 samples with sufficient material [26]. The test is based on the use of fluorescently labeled oligonucleotide probes specific to the centromeric region of chromosome 17 (CEN17) (ZyOrange) and a sequence within the *Her2* gene locus on chromosome 17 (ZyGreen). The slides were processed according to the manufacturer’s protocol: after deparaffinization and rehydration, they were immersed in pre-treatment buffer at 98 °C for 20 min, followed by enzymatic pepsin digestion at 37 °C for 10 min. This was followed by the application of the *Her2/CEN17* probe, subsequent denaturation at 75 °C, and overnight hybridization at 37 °C. Stringent post-hybridization washes and coating with DAPI solution were performed.

The samples were evaluated by fluorescence microscopy (Axiovert S135 microscope, Zeiss) employing filter sets for DAPI, Spec Green (similar to FITC), and Spec Orange (similar to rhodamine), using Diskus Software (Technisches Büro Hilgers, Germany). Thirty tumor nuclei were analyzed per case, and the ratio of green hybridization signals (*Her2*) over red signals (CEP17) was calculated per cell (Her2/Cep17 ratio). According to ASCO guidelines, the specimen was classified as amplified when the Her2/Cep17 ratio was ≥ 2.2, and polysomy 17 was diagnosed when a mean of > 3 CEP17 signals per nucleus was determined, a threshold commonly employed in breast cancer [26].

### Statistical analysis

Differential marker expression between CIS and concomitant invasion was assessed using the Wilcoxon matched-pairs signed-rank test in a group of 48 patients. Also, Her2 and ERβ expression was compared between CIS and normal urothelium in 84 cases with the Wilcoxon matched-pairs signed-rank test. Fisher’s exact test was performed in order to correlate FISH results and Her2 IHC Dako score. The level of significance was set to *p* < 0.05. Analyses were performed using the SPSS Statistics version 20.0 (IBM, USA) and GraphPad Prism 7.0 (GraphPad Software, USA). All figures were generated using GraphPad Prism 7.0.

## Results

Protein expression of luminal (CK20, GATA3, ERβ, Her2) and basal (CK5/6, CK14) markers as well as of p53 was evaluated in 156 CIS specimens from 132 patients (104 men and 28 women) using IHC. The median age of the patient cohort was 70 years (range 42–93 years). In 96/156 specimens (62%), the patient was untreated prior to the biopsy; in 37/156 (24%) cases, the patient had received prior intravesical immunotherapy with BCG; 10/156 (6%) patients had been treated with mitomycin previously; and 4/156 (3%) patients had received both BCG as well as mitomycin before. No pre-treatment data was available in 9/156 (6%) cases. In 37/156 cases (24%), patients presented with pagetoid CIS; in 36/156 cases (23%), the CIS morphology was denuding, and 10/156 (6%) of cases showed both growth patterns (Online Resource 2).

The invasive tumor group consisted of 48 cases of CIS with invasion associated in the same specimen. All invasive tumors were high grade (G3), and 7/48 (15%) were staged pTa, 25/48 (52%) pT1, 15/48 (31%) pT2, and 1/48 was staged pT3 (2%).

### Immunohistochemistry

**Fig. 1:**
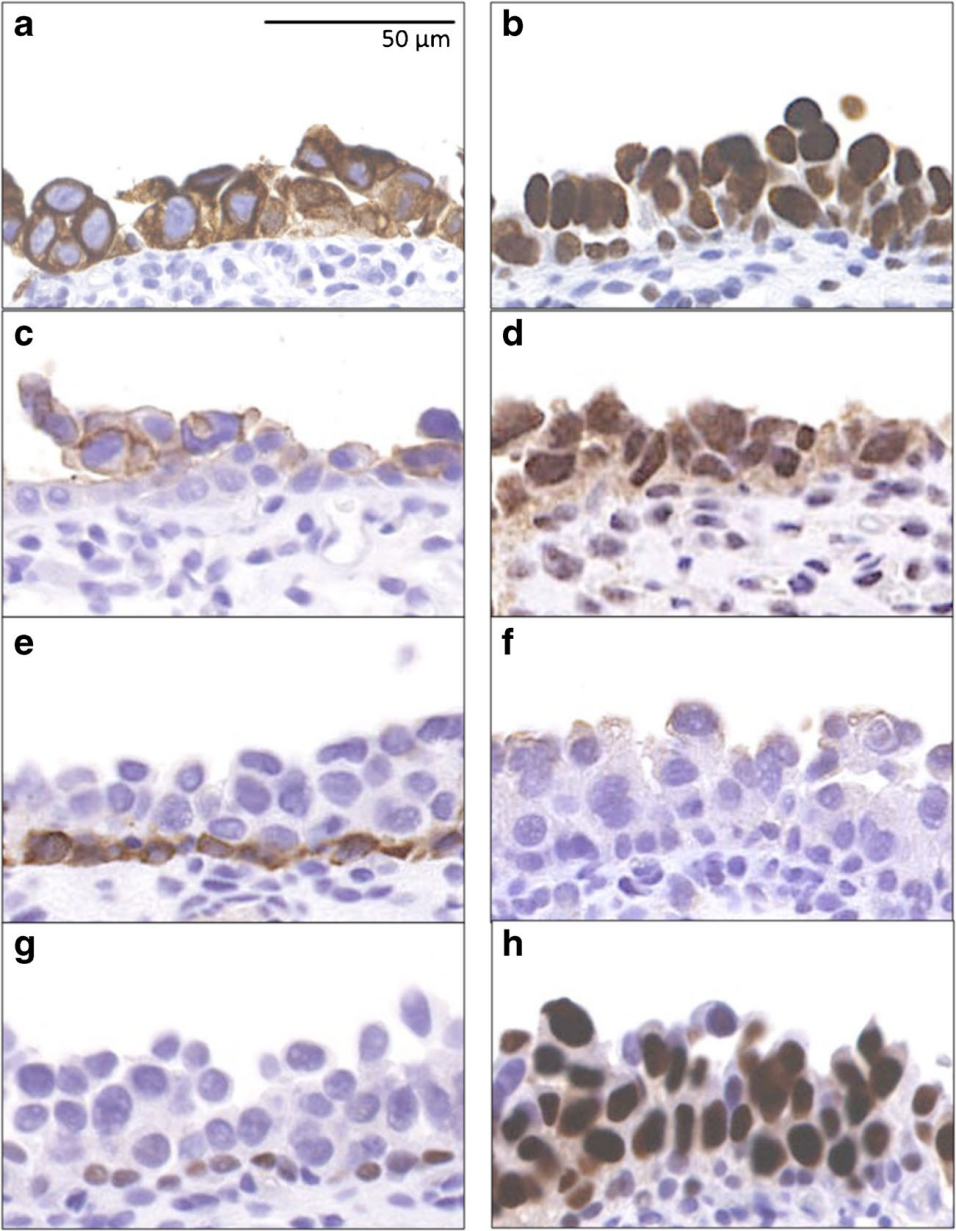
Immunohistochemical staining of carcinoma in situ (CIS) representative of median marker protein expression. **a** Cytokeratin (CK) 20: 85% of cases showed aberrant expression. **b** GATA3: median Remmele score 12. **c** Human epidermal growth factor receptor 2 (Her2) median Dako score 2, with underlying Her2-negative basal cells. **d** Estrogen receptor (ER) β median Remmele score 12. **e** CK5/6 98% of cases negative, with positivity limited to basal cells only. **f** CK14 99% of CIS cases negative. **g** p53-negative CIS (38% of cases). **h** p53-positive CIS (62% of cases). Scale bar 50 μm

The majority of CIS cases was characterized by strong positivity for luminal markers: Aberrant positivity for CK20 was detected in 85% (132/156) of cases, and GATA3 median Remmele score was 12, with 83% (130/156) of cases scored 12 for GATA3 expression. ERβ median Remmele score was 12, and ERβ positivity (defined as Remmele > 2) was seen in 88% (138/156) of CIS specimens. Thirty-two percent of cases (50/156) were scored with a Her2 Dako score of 3+, 33% (51/156) with a Dako score of 2+, whereas only 2% of CIS specimens (3/156) exhibited positivity for CK5/6 and 1% (2/156) for CK14. Aberrant p53 expression was seen in 62% (95/156) of CIS cases (Fig. 1 and Table 1). Expression of the two predictive markers Her2 and ERβ was analyzed in 84 cases of CIS and normal urothelium from the same specimen and was significantly lower in normal urothelium than in CIS (*p* < 0.001) (median Her2 Dako score 1, median ERβ Remmele score 6) (Online Resource 3).

**Table 1:** Protein expression of luminal and basal markers in CIS

	All CIS cases	Median
	*n* = 156 (100%)	
CK20		
Positive	132 (85%)	Positive
Negative	24 (15%)	
Her2		
0–1	55 (35%)	2
2	51 (33%)	
3	50 (32%)	
ERβ		
0–2	18 (12%)	12
3–12	138 (88%)	
GATA3		
0–2	5 (3%)	12
3–12	151 (97%)	
CK5/6		
Positive	3 (2%)	Negative
Negative	153 (98%)	
CK14		
Positive	2 (1%)	Negative
Negative	154 (99%)	

Overview of protein expression of luminal and basal markers in a cohort of 156 CIS cases. Cytokeratin (CK) 20, GATA3, human epidermal growth factor receptor 2 (Her2), and estrogen receptor (ER) β are luminal markers; CK5/6 and CK14 are basal markers. For CK 20, CK5/6, and CK14, percentage of positive cells was determined, and tumors were considered positive if > 50% of cells expressed the CK. GATA3 expression was scored with the Dako score; Her2 and ERβ expression was scored with the Remmele score

**Fig. 2:**
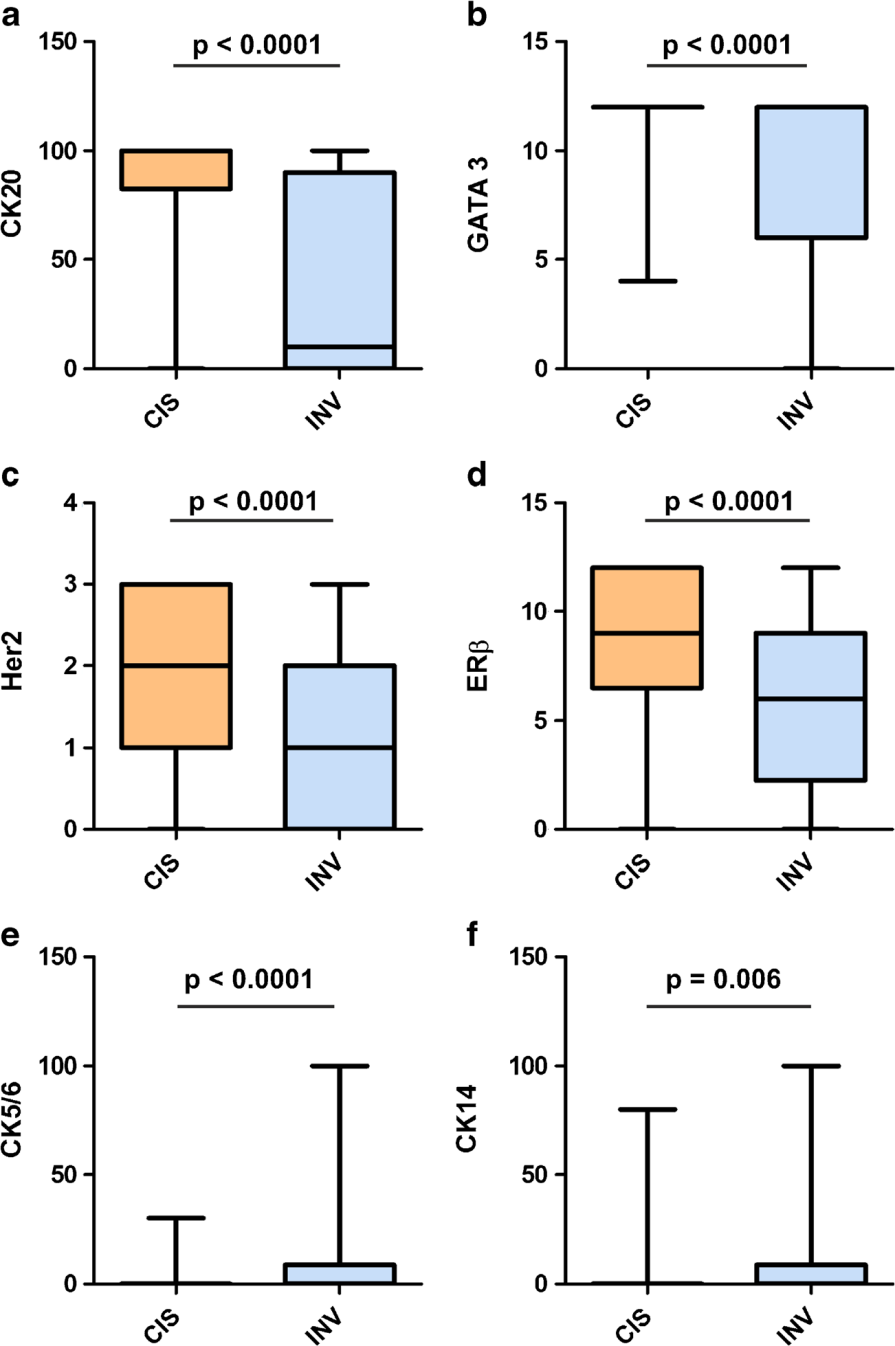
Marker expression of CIS and concomitant invasive tumor. **a** Cytokeratin (CK) 20 (percentage of positive cells evaluated). **b** GATA3 (Remmele Score). **c** Human epidermal growth factor receptor 2 (Her2) (DAKO Score). **d** Estrogen Receptor (ER) β (Remmele Score). **e** CK5/6 (percentage of positive cells). **f** CK14 (percentage of positive cells). Band indicates median, bottom, and top of box show first and third quartiles, whiskers demonstrate range of data distribution (minimum/maximum). A significant downregulation of luminal markers and an upregulation of basal markers were observed in the invasive compartment

We then compared the expression of luminal and basal markers between CIS and corresponding invasive tumor parts from the same biopsy in 48 patients to investigate the stability of marker expression in the course of progression. The expression of all luminal markers was significantly (for all *p* < 0.001) downregulated in the invasive compartment, whereas the reactivity of all basal markers localized almost exclusively to the invasive area (for all *p* < 0.01) (Fig. 2).

### Fluorescence in situ hybridization

**Fig. 3:**
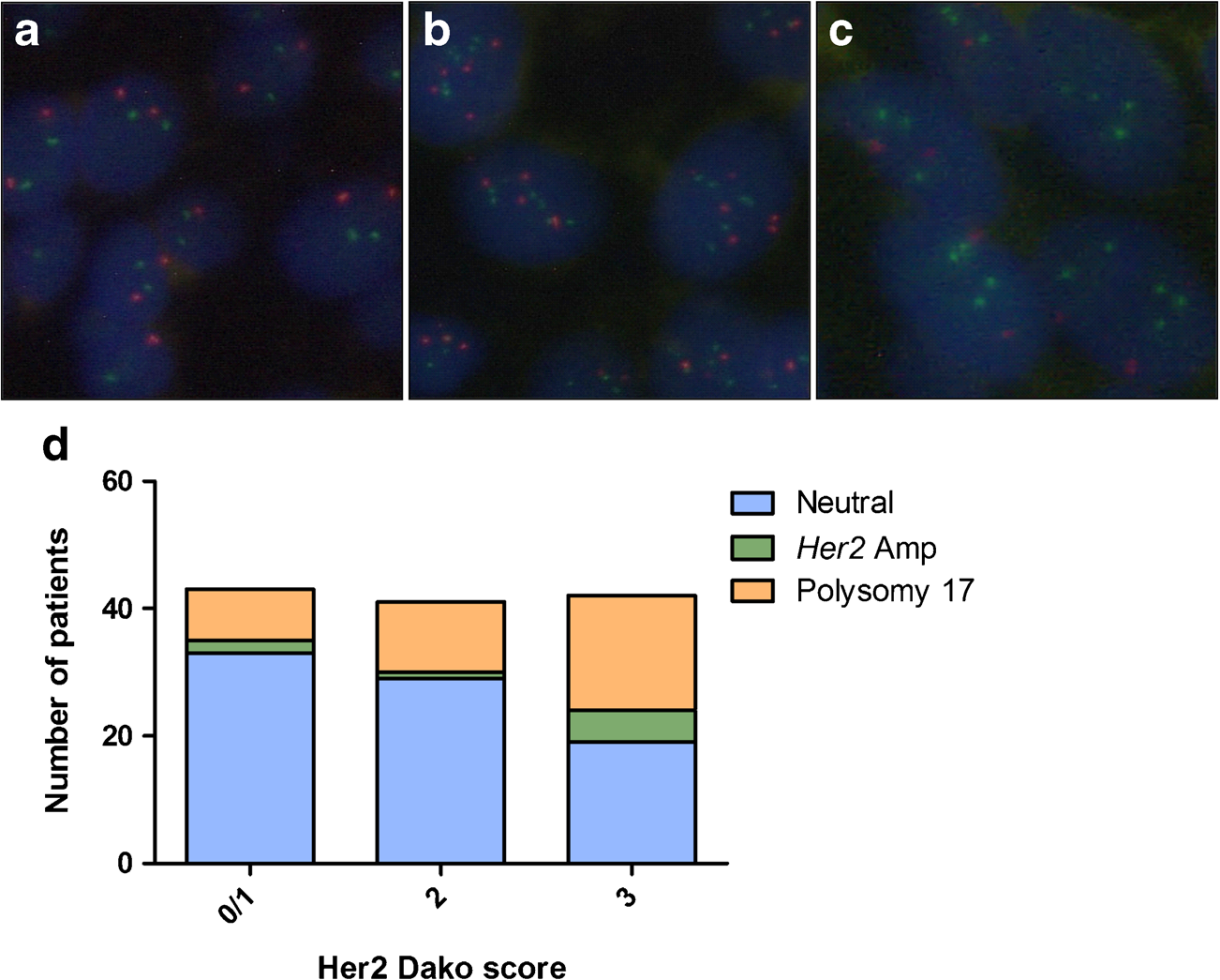
Fluorescence in situ hybridization (FISH). **a** Neutral (non-amplified, non-polysomic). **b** Polysomy 17. **c** *Her2* amplification. **d** Distribution of *Her2*-amplified (Amp), chromosome 17 polysomic, and neutral cases among the various Her2 protein-staining intensities, measured by Dako score. Red hybridization signals indicate the centromeric region of chromosome 17; green signals bind to the *Her2* gene locus on chromosome 17

FISH was performed in 126 FFPE samples to analyze a potential underlying molecular mechanism of Her2 protein expression. *Her2* amplification was detected in 8/126 (6%) cases. Mean CEP17 count indicated polysomy 17 in 37/126 (29%) cases. Her2 protein expression correlated significantly with cases showing polysomy or amplification (Fisher’s exact test *p* = 0.049) (Table 2 and Fig. 3).

**Table 2:** Dako score results for 126 CIS cases analyzed by Her2 FISH

Dako score		0,1+	2+	3+
Neutral	*n* = 81	33	29	19
Polysomy 17	*n* = 37	8	11	18
*Her2*-amplified	*n* = 8	2	1	5

Fluorescence in situ hybridization (FISH) results and immunohistochemical (IHC) staining (Dako score 0–3+) for Her2, showing the distribution of neutral (non-polysomic, non-*Her2*-amplified), chromosome 17 polysomic, and *Her2*-amplified cases in the different IHC categories

## Discussion

In this study, we aimed to elaborate on the affiliation of urothelial CIS to a specific molecular subgroup and to clarify whether molecular subtyping of CIS may also be suitable for prognostic stratification. In this regard, we investigated the immunoreactivity of proteins characteristically expressed in the two intrinsic molecular subsets of bladder cancer [10] and observed that the majority of 156 CIS cases were characterized by the expression of luminal markers and the absence of basal protein expression. In a cohort of 48 patients, we further investigated whether luminal and basal markers remain stable in the course of progression from CIS to invasive disease. Remarkably, we observed that while urothelial CIS strongly and consistently expressed luminal markers, affiliating it to the luminal subtype, this was not the case in the corresponding invasive cells. A significant loss of luminal and a gain of basal marker expression were detected in the invasive compartment.

Data supporting our observation can be extrapolated from recent molecular studies. A strong CIS signature gene expression was found mainly in the basal subgroup of MIBC in the 2017 TCGA dataset, suggesting that these basal tumors evolved from CIS lesions [27]. It was furthermore observed that MIBCs harboring multiple *TP53* and *RB1* pathway alterations, which are also characteristic for the CIS pathway, are found predominantly in the basal subgroup [10, 13].

The hypothesis of CIS as a precursor lesion of basal muscle-invasive tumors is substantiated by the observation that CIS cells parallel the biology of basal urothelial stem cells. Since urothelial stem cells are capable of sustaining long-term growth and proliferation, it is probable that their longevity makes them the most susceptible to the accumulation of oncogenic hits and therefore multistep carcinogenesis [28].

At the same time, molecular evidence underlines the affiliation of CIS to a luminal subgroup. Hedegaard et al. conducted expression profiling of NMIBC, discovering that cases overexpressing the above-mentioned CIS gene signature belonged to the luminal-like group (“class 2”), with high levels of luminal (CK20) and low levels of basal (CK5) markers. As the study only included three CIS cases, we consider our significantly larger cohort a possible endorsement of these data [14]. The underlying mechanisms causing CIS cells to consistently express luminal markers, as well as the stimuli inducing the basal protein expression in invasive cells of CIS origin, remain to be explored.

Only minor advances towards more efficient and targeted intravesical therapies for urothelial CIS have been made in the last decades [29]. At the time of initial diagnosis, an organ-sparing approach of transurethral resection and BCG instillation is usually chosen [30]. The latter in particular is associated with severe morbidity and a high probability of so-called BCG failure, including disease progression and recurrence [31]. The risk of tumor progression to a life-threatening muscle-invasive situation encourages clinicians to recommend radical cystectomy in patients with extensive or recurrent CIS. This procedure is on the one hand associated with an excellent tumor-specific survival [32], but on the other hand bears a significant risk of overtreatment and associated surgical morbidity [8, 33]. Clinical and preclinical work on new therapeutic targets in bladder cancer has mainly focused on MIBC [34], and few authors have examined new therapeutic targets in urothelial CIS [29].

Two markers from our luminal panel, Her2 and ER, are well-established predictive markers and therapeutic targets in other tumor entities [35, 36]. We aimed to evaluate the therapeutic potential of Her2 and ERβ in urothelial CIS by assessing protein expression of both markers, as well as the molecular background of Her2 expression by FISH analysis.

Two ERs exist in the human body, ERα and β, which mediate ligand-dependent transactivation of gene expression [37]. Not only is ERβ the predominant ER in the bladder with a potential prognostic role in NMIBC, but it is also postulated to promote cancer growth and progression [16]. Multiple studies have investigated ERβ protein expression in both normal urothelium as well as urothelial carcinoma [16, 17, 38]; however, to the best of our knowledge, none of these included CIS. Our analyses showed ERβ positivity in 88% (138/156) of CIS cases.

ERβ has previously been identified as a druggable target in bladder cancer using the selective ER modulator Raloxifene or ERβ-specific antagonists such as PHTPP [16, 35]. Furthermore, current evidence suggests that ERβ plays a central role in the proliferative and invasive potential of bladder cancer cells and therefore may present a promising target for selective ERβ inhibitors [39]. As such, the observed ERβ positivity in the majority of CIS cases in our cohort warrants further investigation of ERβ as a potential target in early, flat, high-grade bladder cancer.

The other marker with therapeutic potential included in our IHC panel is Her2, a member of the epidermal growth factor receptor family, which, when overexpressed, enhances proliferation, cell survival, and the invasive capacity of tumor cells [40, 41]. Anti-Her2 therapy has emerged as the mainstay of treatment in Her2-amplified breast and gastric cancer [18, 20]. Conflicting findings have been reported on the rate and the mechanism of Her2 overexpression in MIBC, which, after breast and gastric cancer, shows the highest rate of *Her2* amplification [13]. A recent investigation of the mechanism of Her2 expression in MIBC showed that Her2 protein overexpression arises from various mechanisms, including gene amplification [42]. Though Her2 immunostaining has previously been investigated as a diagnostic tool for urothelial CIS [43], our study is the first to examine Her2 expression in a significantly larger patient cohort and the first to investigate the possible genetic background of Her2 expression in CIS. IHC characterized 52 cases (33%) as Her2 moderate (2+) and 50 cases (32%) as Her2 positive (3+).

FISH analysis detected only a low rate of *Her2* amplifications in 8/126 cases (6%). At the same time, we observed polysomy 17, indicated by an elevated CEP17 count, in 37/126 (29%) of cases. We observed a significantly higher Her2 expression in cases with polysomy 17 or Her2 amplification, compared to CIS cases without these alterations. Chromosome 17 polysomy has been previously described as one of the mechanisms driving Her2 expression in breast cancer, especially in tumors with IHC 2+ scores [44]. Interestingly, various studies support the effectivity of trastuzumab, a humanized anti-HER2 monoclonal antibody, in polysomic, non-amplified breast cancer [45, 46].

Although none are currently in clinical use for bladder cancer, there is accumulating evidence that anti-Her2-targeted therapies are promising novel treatment strategies in urothelial carcinoma [42]. As the driver status of Her2, even in Her2-overexpressing or Her2-amplified bladder cancer cases, is difficult to predict without detailed genome-wide analyses [42], we propose the use of agents whose impact is independent of Her2-signaling inhibition. Targeting Her2-overexpressing cells via antibody-mediated cytotoxicity of clinically established monoclonal antibodies like trastuzumab or the cytotoxic effects of the emerging antibody-drug conjugate trastuzumab-DM1 (T-DM1) may be a viable option [36, 47]. We see particular potential for clinical studies investigating the response rate of an intravesical combination therapy of BCG and T-DM1. Furthermore, the large number of patients with BCG-refractory CIS may benefit from a Her2 targeting, which would provide a bladder-sparing approach for patients otherwise subjected to cystectomy.

We are aware of the limitations of this retrospective study. Firstly, the observations from this study need to be validated in independent patient cohorts, possibly with an expansion to other superficial urothelial cancers, such as non-invasive papillary high-grade tumors. Secondly, further molecular studies are needed to explain the phenomenon of the observed marker switch from luminal to basal in the course of invasion. Thirdly, the possible targets identified in this study, ERβ and Her2, belong to the luminal marker panel, which is downregulated in the process of invasion, possibly limiting the suitability of ERβ and Her2 targeting to CIS and should encourage combined therapy modalities.

None withstanding the aforementioned limitations, we have for the first time reported on a shift from luminal to basal marker expression in urothelial carcinoma and distinguished two potential therapeutic targets in CIS. In summary, positivity for either ERβ, Her2, or both proteins was observed in 91% (142/156) of CIS cases, while normal urothelium showed significantly lower expression of both markers, highlighting their potential for clinical use. The protein targets identified in this study, Her2 and ERβ, may be amenable to targeted intravesical therapies in early-stage, high-grade bladder cancer. As mentioned above, validation in an independent cohort of patients and clinical trials are required to confirm our preliminary findings.

## Electronic supplementary material

ESM 1 (PDF 24 kb)

ESM 2 (PDF 461 kb)

ESM 3 (PDF 515 kb)

## Abbreviations

BCG: Bacillus Calmette-Guérin
CIS: Carcinoma in situ
CK: Cytokeratin
CUETO: Spanish Urological Club for Oncological Treatment
EORTC: European Organization for Research and Treatment of Cancer
ER: Estrogen receptor
FFPE: Formalin-fixed paraffin-embedded
FISH: Fluorescence in situ hybridization
Her2: Human epidermal growth factor receptor type 2
IHC: Immunohistochemistry
MIBC: Muscle-invasive bladder cancer
NMIBC: Non-muscle-invasive bladder cancer
TMA: Tissue microarray
T-DM1: Trastuzumab-DM1
