# Supplementary material for: Progression of urothelial carcinoma in situ of the urinary bladder: a switch from luminal to basal phenotype and related therapeutic implications
Source: Virchows Arch. 2018 Apr 13;472(5):749–58. doi: 10.1007/s00428-018-2354-9 (PMC5978840; doi:10.1007/s00428-018-2354-9)
Supplement: Supplementary file 1 — (PDF 24 kb) [file 428_2018_2354_MOESM1_ESM.pdf]

Online Resource 1 – Virchows Archiv

Progression of urothelial carcinoma *in situ* of the urinary bladder: a switch from luminal to basal phenotype and related therapeutic implications

Isabella Barth, Ursula Schneider, Tobias Grimm, Alexander Karl, David Horst, Nadine T. Gaisa, Ruth Knüchel and Stefan Garczyk

Corresponding author:

Prof. Dr. med. Ruth Knüchel
Institute of Pathology
University Hospital RWTH Aachen
Pauwelsstrasse 30, 52074 Aachen, Germany
Email: rknuechel-clarke@ukaachen.de

Antibody	Source	Clone/ Reference	Dilution (FFPE; Frozen)
CK20	Dako	Ks20.8	1/100; 1/200
GATA3	Biocare Medical	CM405A	1/250; 1/250
Her2	Dako	Polyclonal	1/300; 1/500
ERβ	Dako	PPG5/10	1/20; 1/20
CK5/6	Dako	D5/16 B4	1/100; 1/100
CK14	Abcam	ab7800	1/400; 1/200
p53	Dako	318-6-11	1/50; 1/50

Online resource 1: Overview of antibodies

Abbreviations: CK – Cytokeratin, ER – Estrogen Receptor, FFPE – Formalin-fixed, paraffin embedded, Her2 – Human epidermal growth factor receptor 2
