# Supplementary material for: Progression of urothelial carcinoma in situ of the urinary bladder: a switch from luminal to basal phenotype and related therapeutic implications
Source: Virchows Arch. 2018 Apr 13;472(5):749–58. doi: 10.1007/s00428-018-2354-9 (PMC5978840; doi:10.1007/s00428-018-2354-9)
Supplement: Supplementary file 2 — (PDF 461 kb) [file 428_2018_2354_MOESM2_ESM.pdf]

Online Resource 2 – Virchows Archiv

# Progression of urothelial carcinoma *in situ* of the urinary bladder: a switch from luminal to basal phenotype and related therapeutic implications

Isabella Barth, Ursula Schneider, Tobias Grimm, Alexander Karl, David Horst, Nadine T. Gaisa, Ruth Knüchel and Stefan Garczyk

Corresponding author:

Prof. Dr. med. Ruth Knüchel
Institute of Pathology
University Hospital RWTH Aachen
Pauwelsstrasse 30, 52074 Aachen, Germany
Email: rknuechel-clarke@ukaachen.de

| | all CIS cases n=156 | (100%) |
|---|---|---|
| pre-treatment | | |
| previously untreated CIS | 96 | (62%) |
| previous BCG treatment | 37 | (24%) |
| previous Mitomycin treatment | 10 | (6%) |
| previous BCG and Mitomycin treatment | 4 | (3%) |
| no data available | 9 | (6%) |
| type of biopsy | | |
| TUR | 146 | (94%) |
| Cystectomy | 10 | (6%) |
| Variant CIS growth pattern | | |
| pagetoid CIS cell spread | 37 | (24%) |
| denuding morphology | 36 | (23%) |
| pagetoid and denuding growth pattern | 10 | (6%) |
| no pagetoid or denuding growth pattern | 73 | (47%) |

**Online resource 2: Clinicopathological data**

Abbreviations: BCG – Bacillus Calmette-Guérin, CIS – Carcinoma in situ, TUR – Transurethral resection
