# Supplementary material for: Progression of urothelial carcinoma in situ of the urinary bladder: a switch from luminal to basal phenotype and related therapeutic implications
Source: Virchows Arch. 2018 Apr 13;472(5):749–58. doi: 10.1007/s00428-018-2354-9 (PMC5978840; doi:10.1007/s00428-018-2354-9)
Supplement: Supplementary file 3 — (PDF 515 kb) [file 428_2018_2354_MOESM3_ESM.pdf]

Online Resource 3 – Virchows Archiv

Progression of urothelial carcinoma *in situ* of the urinary bladder: a switch from luminal to basal phenotype and related therapeutic implications

Isabella Barth, Ursula Schneider, Tobias Grimm, Alexander Karl, David Horst, Nadine T. Gaisa, Ruth Knüchel and Stefan Garczyk

Corresponding author:

Prof. Dr. med. Ruth Knüchel
Institute of Pathology
University Hospital RWTH Aachen
Pauwelsstrasse 30, 52074 Aachen, Germany
Email: rknuechel-clarke@ukaachen.de

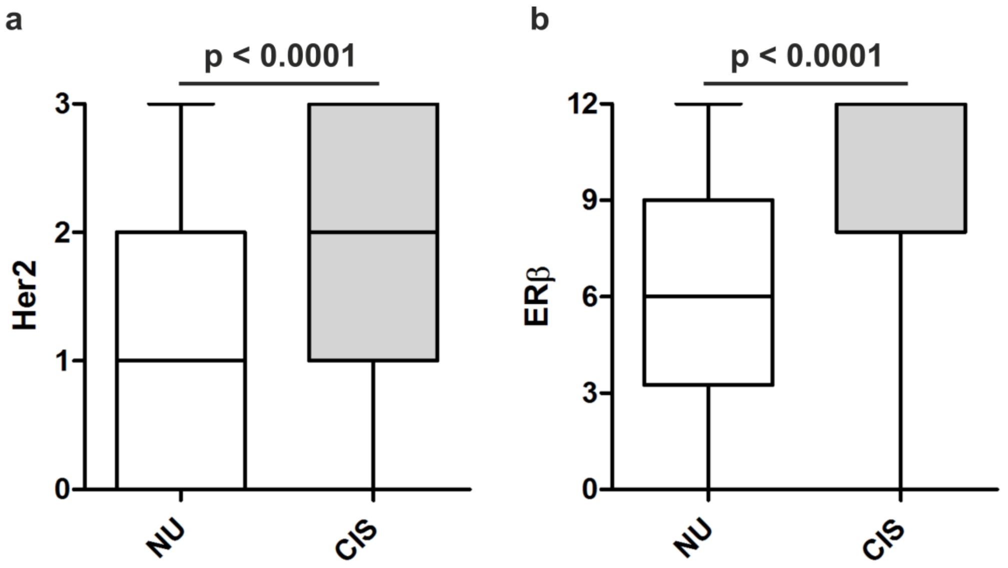

Online resource 3: Comparison of Her2 and ERβ expression in CIS and NU
(a) Her2 expression (Dako score) and **(b)** ERβ expression (Remmele Score) were compared in CIS and normal urothelium at the same time of biopsy in 84 cases with available normal urothelium. A significantly higher expression of both proteins was observed in the CIS cohort, highlighting the potential of intravesical anti-Her2 and anti-ERβ therapies.

Abbreviations: CIS – Carcinoma in situ, ER – Estrogen receptor, Her2 – Human epidermal growth factor receptor 2, NU – normal urothelium
